# Comprehensive Analysis of CD4^+^ T Cell Response Cross-Reactive to SARS-CoV-2 Antigens at the Single Allele Level of HLA Class II

**DOI:** 10.3389/fimmu.2021.774491

**Published:** 2022-01-06

**Authors:** You-Seok Hyun, Yong-Hun Lee, Hyeong-A Jo, In-Cheol Baek, Sun-Mi Kim, Hyun-Jung Sohn, Tai-Gyu Kim

**Affiliations:** ^1^ Department of Microbiology, College of Medicine, The Catholic University of Korea, Seoul, South Korea; ^2^ Department of Biomedicine and Health Sciences, College of Medicine, The Catholic University of Korea, Seoul, South Korea; ^3^ Catholic Hematopoietic Stem Cell Bank, College of Medicine, The Catholic University of Korea, Seoul, South Korea

**Keywords:** CD4^+^ T cells, cross-reactive T cells, SARS-CoV-2, MHC class II alleles, HLA-DR, HLA-DQ, HLA-DP, immunodominance

## Abstract

Common human coronaviruses have been circulating undiagnosed worldwide. These common human coronaviruses share partial sequence homology with severe acute respiratory syndrome coronavirus 2 (SARS-CoV-2); therefore, T cells specific to human coronaviruses are also cross-reactive with SARS-CoV-2 antigens. Herein, we defined CD4^+^ T cell responses that were cross-reactive with SARS-CoV-2 antigens in blood collected in 2016–2018 from healthy donors at the single allele level using artificial antigen-presenting cells (aAPC) expressing a single HLA class II allotype. We assessed the allotype-restricted responses in the 42 individuals using the aAPCs matched 22 HLA-DR alleles, 19 HLA-DQ alleles, and 13 HLA-DP alleles. The response restricted by the HLA-DR locus showed the highest magnitude, and that by HLA-DP locus was higher than that by HLA-DQ locus. Since two alleles of HLA-DR, -DQ, and -DP loci are expressed co-dominantly in an individual, six different HLA class II allotypes can be used to the cross-reactive T cell response. Of the 16 individuals who showed a dominant T cell response, five, one, and ten showed a dominant response by a single allotype of HLA-DR, -DQ, and -DP, respectively. The single allotype-restricted T cells responded to only one antigen in the five individuals and all the spike, membrane, and nucleocapsid proteins in the six individuals. In individuals heterozygous for the HLA-DPA and HLA-DPB loci, four combinations of HLA-DP can be expressed, but only one combination showed a dominant response. These findings demonstrate that cross-reactive T cells to SARS-CoV-2 respond with single-allotype dominance.

## Introduction

Severe acute respiratory syndrome coronavirus 2 (SARS-CoV-2) infection causes coronavirus disease 2019 (COVID-19). SARS-CoV-2 is clustered with SARS-CoVs of the genus *Betacoronavirus* belonging to the family Coronaviridae (1). SARS-CoV-2 consists of a lipid bilayer containing spike proteins and RNA genome bound by nucleocapsid proteins and condensed by membrane proteins (2, 3). The spike protein binds to human angiotensin-converting enzyme 2 (hACE2), and SARS-CoV-2 enters hACE2 expressing cells *via* endocytosis (4). If a neutralizing antibody is generated to block the receptor-binding domain (RBD) of the spike protein, then the SARS-CoV-2 infection can be prevented (5).

SARS-CoV-2-specific adaptive immunity limits COVID-19 disease severity by coordinating SARS-CoV-2-specific CD4^+^ and CD8^+^ T cells (6). An mRNA or adenovirus-vector encoding spike protein has been developed and is used to vaccinate individuals who have been mainly unexposed to SARS-CoV-2. These vaccines induce virus-neutralizing antibody, spike protein-specific CD8^+^ T cell, and CD4^+^ T cell responses, boosting the humoral and cellular responses (7, 8). HLA-DR, -DQ, and -DP loci are human leukocyte antigen (HLA) class II loci, which are the MHC class II genes in humans and are recognized by CD4^+^ T cells. The loci of HLA genes are highly polymorphic, with the polymorphic residues primarily in the peptide binding grooves (9). The peptide repertoire presented by the HLA allotypes varies so that some HLA alleles are associated with the susceptibility of the given pathogens (10–12).

For influenza-specific CD4^+^ T cell response, T cells cross-recognize multiple strains by targeting highly conserved viral proteins, mainly internal proteins constrained by functional limitations of viral fitness (13). New SARS-CoV-2 variants have emerged, some of which harbor mutations in the spike protein and RBD. The neutralizing antibody titer, induced by vaccination or SARS-CoV-2 infection, was lower in the variants than in the wild type (14). However, there were no differences in the CD4^+^ T cell response to the variants and wild type, and the sequences of SARS-CoV-2 T cell epitopes were not affected by the mutations found in the variants (15–17).

Among the family Coronaviridae, there are four common human coronaviruses (HCoV-299E, HCoV-NL63, HCoV-OC34, and HCoV-HKU1s) that cause mild upper respiratory illness and have been circulating undiagnosed worldwide (18). These HCoVs share partial sequence homology with SARS-CoV-2; therefore, T cells specific to the HCoVs cross-reactively responded to SARS-CoV-2 (19, 20). The cross-reactive T cells enhanced vaccine antibody responses and were suggested to be associated with milder symptoms, and T cells were indispensable for protection against SARS-CoV-2 infection in rhesus macaques (21–23). In the present study, we assessed CD4^+^ T cell responses that are cross-reactive with SARS-CoV-2 antigens in blood collected in 2016–2018 from healthy donors at a single allele level using artificial antigen-presenting cells (aAPCs) expressing a single HLA class II allele.

## Materials and Methods

### Human Blood Samples

This study was approved by the Institutional Review Board of the Catholic University of Korea (MC21SASI0009). All healthy donors provided written informed consent prior to participation in this study. The participants are ranged from 26.9 ± 5.0 years of age and composed of 4 females and 38 males. Peripheral blood mononuclear cell (PBMC) samples were obtained from 350 ml of leukapheresis products of the donors. PBMCs were further purified from the leukapheresis product by density gradient centrifugation using Ficoll–Hypaque (GE Healthcare). CD4^+^ T cells were isolated using magnetic microbeads (AutoMacs Pro separator; Miltenyi Biotec), and the purity of the CD4^+^ T cells was confirmed with flow cytometry (97% ± 2%). Suspended CD4^+^ T cells in fetal bovine serum (Gibco) containing 10% dimethyl sulfoxide (Mylan) and 50% RPMI 1640 medium (Lonza) were cryopreserved in liquid nitrogen until incubation with aAPCs for ELISPOT assay.

The genomic DNA was isolated from the red blood cells and granulocytes (Tiangen Biotech Corporation). The HLA was then genotyped using polymerase chain reaction sequencing-based typing and next-generation sequencing (**Table 1**) (NCBI BioProject Accession: PRJNA721949) (MiSeqDx, Illumina), as previously described (24, 25). The resolution of the HLA types was converted to 4-digits from 6-digits to analyze the HLA molecules at the protein level.

### Generation of aAPCs Expressing Single HLA Class II Allotype

For cloning, cDNAs were isolated from the lymphoblastoid cell lines typed each HLA allele (740902.50; Macherey Nagel, RT300M; Enzynomics). Unlike DRB1, there was polymorphism near the start and stop codon on DQA1, DQB1, DPA1, and DPB1 loci; therefore, each DQ and DP allele was amplified using a primer containing a short consensus sequence at the coding region and the sequence at 5′UTR or 3′UTR with mixed bases (**Table S1**). HLA class II alleles were then cloned into the pCDH lentivector (#CD523A-1; System Biosciences) using In-Fusion Cloning (EZ015TL; Enzynomics).

As previously described (26), 5 × 10^6^ 293TN producer cells (System Biosciences) were transfected (Lipofectamine 2000; Invitrogen) with 1.3 pmol psPAX2 (RRID: Addgene_12260), 0.72 pmol pMD.2G (RRID: Addgene_12259), and 1.64 pmol single HLA class II allele-encoding pCDH, and the supernatant was harvested after 2 days. The K562-based aAPCs, which do not express HLA molecules, were transduced with lentiviruses encoding an alpha and a beta chain at a multiplicity of infection (MOI) of 20. The transduced aAPCs were purified by fluorescence-activated cell sorting (FACSAria Fusion, BD Biosciences). For the sorting, the following monoclonal antibodies were used (**Table S2**): HLA-DR (clone G46-6; RRID: AB_1727527), HLA-DQ (clone Tü169; RRID: AB_2738963, Tü39; RRID: AB_395940), and HLA-DP (clone B7/21). The aAPCs were not stained with those antibodies when transduced with only an alpha chain or a beta chain. The aAPCs were then cultured in RPMI 1640 supplemented with 2 mM L-glutamine, 100 U/ml penicillin–streptomycin–amphotericin B mixture (Lonza), and 10% fetal bovine serum (Gibco). We confirmed HLA class II expression using flow cytometry (FACSCanto, BD Biosciences) before cryopreservation of the aAPCs.

### Measurement of Single HLA Class II Allotype-Restricted CD4^+^ T Cell Response by *Ex Vivo* IFN-γ ELISPOT Assay

The single HLA class II allotype-expressing aAPCs were thawed in complete media and seeded at 5 × 10^4^ cells per a well in a 96-well cell culture plate. The aAPCs were resuspended in serum-free media and pulsed with 60 nM of each peptide pool (JPT Peptide Technologies) for 2 h at 37°C in 5% CO_2_. The antigen-loaded aAPCs were washed three times with serum-free media. The peptide-pulsing and washing were performed using centrifugation and a microplate washer (405LSR; BioTek) in a 96-well cell culture plate. The 5 × 10^5^ CD4^+^ T cells were incubated with 5 × 10^4^ aAPCs in an ELISPOT plate for 20 h at 37°C in 5% CO_2_ (551849; BD Biosciences). The 4 × 10^6^ to 7 × 10^6^ and 1.8 × 10^7^ to 2.1 × 10^7^ CD4^+^ T cells per donor were used for measuring SARS-CoV-2 cross-reactive CD4^+^ T cell response by an allotype and to a SARS-CoV-2 antigen, respectively. The 5 × 10^6^ CD4^+^ T cells per donor were used for measuring the response by a combination of HLA-DP heterodimer. The spot forming cells (SFC) were counted using an AID ELISPOT Reader System (AID Diagnostika GmbH). The magnitude of an allotype-restricted CD4^+^ T cell response to antigens was calculated as [(response to aAPCs expressing HLA pulsed with peptide pools) − (response to aAPCs expressing HLA)] − [(response to aAPCs pulsed with peptide pools) − (response to aAPCs)], as previously described (26, 27). The ELISPOT results were presented as SFC per million CD4^+^ T cells.

### Data Processing and Statistical Analysis

The Google Spreadsheets were used for calculating the magnitude of response by a given allotype, sorting the order of highest response, and filtering the magnitude of response by a locus and an allele. The data were visualized in Microsoft Excel, GraphPad Prism 7, and FlowJo v10 (BD). Statistical analyses were performed by GraphPad Prism 7 software. Statistical significance was determined by one-way analysis of variance [ANOVA], Pearson’s correlation analysis, Welch’s t-test (with a two-tailed test of significance). Values of P <0.05 were considered significant. The data are expressed as means ± standard deviation or standard error of the mean, and the sample sizes are presented in the figures.

## Results

### SARS-CoV-2 Cross-Reactive CD4^+^ T Cell Responses According to HLA Class II Loci and Alleles

The peptide binding grooves of HLA class I are only in the alpha chain, whereas the alpha and beta chains of HLA class II form heterodimers, presenting the epitope by a matching pair of an allotype (28). HLA-DRB1, -DQA1, -DQB1, -DPA1, and -DPB1 alleles were genotyped in 42 donors to assess the CD4^+^ T cell responses in an allotype-specific manner (**Table 1**). The DRA locus encoding the alpha chain of HLA-DR is almost monomorphic; however, the DQA and DPA loci encoding the alpha chain of HLA-DQ and -DP are polymorphic. We further estimated the most likely haplotype for HLA-DQ and DP within individuals based on haplotype frequency. We established single HLA-DR, -DQ, and -DP allotype-expressing aAPCs of a haplotypic pair in 42 donors in addition to those previously established (**Figure S1**) (26).

**Table 1:** Genotypes of HLA class II in 42 healthy donors.

Donor	Age	Sex	DRB1*	DRB1*	DQA1*/DQB1*	DQA1*/DQB1*	DPA1*/DPB1*	DPA1*/DPB1*
HD01	24	M	13:02	15:01	02:01/02:02	03:01/03:02	02:01/13:01	02:02/05:01
HD02	32	M	01:01	15:01	01:01/05:01	01:02/06:02	01:03/02:02	02:02/02:02
HD03	33	M	04:10	08:03	03:03/04:02	06:01/03:01	02:01/14:01	
HD04	24	M	08:03	14:05	01:03/06:01	01:04/05:03	02:02/05:01	
HD05	27	M	01:01	09:01	01:01/05:01	03:02/03:03	01:03/04:02	02:02/05:01
HD06	21	M	04:05	04:06	03:01/03:02	03:03/04:01	01:03/04:02	02:02/05:01
HD07	26	M	04:06	09:01	03:01/03:02	03:02/03:03	02:02/05:01	
HD08	19	M	08:02	14:54	01:04/05:02	03:01/03:02	01:03/04:02	02:02/05:01
HD09	30	M	04:06	08:03	03:01/03:02	06:01/03:01	01:03/02:01	02:02/05:01
HD10	31	M	04:03	13:02	03:01/03:02	01:02/06:04	02:02/05:01	01:03/04:01
HD11	25	M	01:01	14:05	01:01/05:03	01:01/05:01	01:03/02:01	
HD12	32	M	01:01	15:02	01:01/05:01	01:03/06:01	01:03/04:02	01:03/03:01
HD13	27	M	07:01	12:01	02:01/02:02		01:03/04:02	02:01/17:01
HD14	30	M	04:05	07:01	02:01/02:02	03:03/04:01	01:03/02:01	02:02/05:01
HD15	31	M	12:01		03:01/03:02	01:01/05:01	02:02/05:01	02:01/09:01
HD16	28	M	12:02	15:01	01:02/06:02	06:01/03:01	02:02/05:01	
HD17	24	M	04:06	15:01	01:02/06:02	03:01/03:02	01:03/02:01	02:02/03:01
HD18	26	M	04:06	14:05	01:01/05:03	03:01/03:02	01:03/02:01	02:02/05:01
HD19	26	M	04:05	14:05	01:04/05:03	03:03/04:01	02:02/05:01	
HD20	22	M	08:02	14:06	03:01/03:02	05:03/03:01	02:01/13:01	02:02/05:01
HD21	32	M	07:01	09:01	02:01/02:02		01:03/05:01	02:01/13:01
HD22	24	M	08:03	12:01	01:03/06:01		01:03/03:01	01:03/04:01
HD23	26	F	12:01	15:01	01:02/06:02		01:03/02:01	
HD24	28	M	08:03	15:01	01:02/06:02	01:03/06:01	01:03/04:02	02:02/05:01
HD25	45	M	13:02	16:02	01:02/06:09	01:02/05:02	01:03/02:02	02:02/05:01
HD26	23	M	04:06	09:01	03:01/03:02	03:02/03:03	02:01/14:01	02:02/05:01
HD27	33	M	04:04	09:01	03:02/03:03		01:03/04:02	02:02/05:01
HD28	24	M	08:03		01:03/06:01		01:03/04:02	02:02/05:01
HD29	25	M	04:05		03:03/04:01		01:03/04:02	
HD30	23	M	04:06	14:05	01:04/05:03	03:01/03:02	02:02/03:01	02:02/05:01
HD31	28	M	04:03	12:01	03:01/03:02	05:05/03:01	01:03/02:01	
HD32	27	M	04:06	14:54	01:04/05:03	03:01/03:02	02:01/13:01	02:02/05:01
HD33	25	M	01:01	08:03	01:01/05:01	01:03/06:01	01:03/02:01	02:02/05:01
HD34	26	M	04:04	08:03	03:01/03:02	01:03/06:01	02:02/02:02	02:01/09:01
HD35	20	F	01:01	08:03	01:01/05:01	01:03/06:01	01:03/04:02	02:02/05:01
HD36	31	M	03:01	10:01	05:01/02:01		01:03/04:01	02:01/09:01
HD37	22	M	07:01	15:01	02:01/02:02	05:05/03:01	02:02/05:01	02:01/17:01
HD38	24	F	01:01	13:02	01:01/05:01	01:02/06:04	02:02/05:01	
HD39	20	M	07:01	14:05	01:04/05:03	02:01/02:02	01:03/02:02	02:02/05:01
HD40	30	M	13:02	15:01	01:02/06:02	01:02/06:09	02:02/03:01	02:02/05:01
HD41	36	M	07:01	14:54	01:04/05:02	02:01/02:02	01:03/02:01	02:01/17:01
HD42	19	F	04:01	14:54	01:04/05:02	03:03/03:01	01:03/02:01	01:03/04:02

CD4^+^ T cells were stimulated with an allotype-matched aAPCs pulsed with a mixture of peptide pools of the spike, nucleocapsid, and membrane proteins. CD4+ T cells were co-cultured with unpulsed allotype-matched aAPCs and aAPCs untransduced HLA as a control. SARS-CoV-2 cross-reactive CD4^+^ T cell responses by an allotype were measured using the IFN-γ ELISPOT assay and calculated as described in *Materials and Methods*, data shown in **Table S3**. The response of isolated CD4^+^ T cells to aAPCs, which were untransduced with the HLA-specific lentivirus, was 1.5 ± 2.9 SFCs. The response to peptide pools- pulsed aAPCs was 1.7 ± 2.9 SFCs.

**Figure 1:**
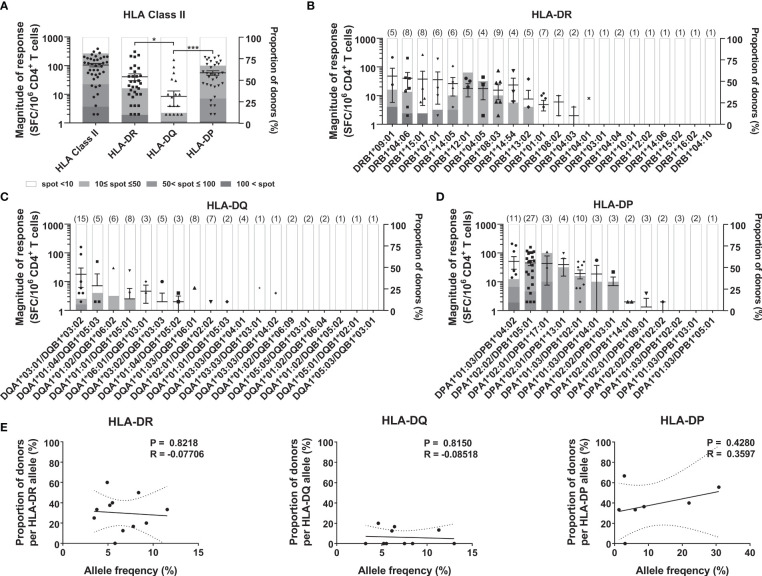
CD4^+^ T cell responses to SARS-CoV-2 antigens according to HLA class II loci and alleles. CD4^+^ T cells were stimulated with an allotype-matched aAPCs pulsed with a mixture of peptide pools of spike, nucleocapsid, and membrane protein. The responses by HLA-DR, -DQ, and -DP loci **(A)**, and HLA-DR alleles **(B)**, HLA-DQ alleles **(C)**, and HLA-DP alleles **(D)** in 42 donors were compared. Each dot presents the magnitude of response by a locus or an allele in an individual. Error bars indicate the mean ± standard error of the mean (SEM), and the number of donors with the allele are shown in parentheses. The stacked bar graph presents a proportion of the classified response in donors out of the donors with the allele. Statistical analysis was performed using a one-way ANOVA. *P < 0.05, ***P < 0.001. **(E)** Correlation of allele frequency with the proportion of positive response by HLA-DR, -DQ, and -DP alleles that are in at least three individuals. Statistical analysis was performed using Pearson’s correlation analysis. A line of best fit (solid lines) and the 95% confidence bands (dotted lines) were analyzed using linear regression analysis.

The magnitude of response by a locus was calculated by summing the responses of two alleles in each HLA-DR, -DQ, and -DP locus within an individual to understand which HLA class II locus was preferentially used in the response. Similar to the CMV pp65-specific CD4^+^ T cell response (26), the magnitude of response by the HLA-DR locus was significantly higher than that by the HLA-DQ locus (**Figure 1A**, one-way ANOVA, *p* = 0.0359). The magnitude of response of the HLA-DP locus was significantly higher than that of the HLA-DQ locus (**Figure 1A**, one-way ANOVA, *p* = 0.0002). We classified the magnitude of response into stepwise responses with more than 100, 50, or 10 SARS-CoV-2-specific cells per 5 × 10^5^ CD4^+^ T cells: strong, moderate, and positive response, respectively. For the HLA-DR locus, 9.5% of the donors showed a strong response and no donors showed a moderate response (**Figure 1A**). For the HLA-DQ locus, no donors showed a strong response, and 2.4% showed a moderate response. For the HLA-DP locus, 2.4% of the donors showed a strong response, and 26.2% showed a moderate response. When the response of the HLA class II was calculated by summing the responses of the HLA-DR, -DQ, and -DP loci within an individual, the magnitude of SARS-CoV-2 cross-reactive CD4^+^ T cell response was 53/5 × 10^5^, which was lower than that of the CMV pp65-specific CD4^+^ T cell responses (267/5 × 10^5^).

Next, we analyzed the response of each allotype to determine which allele on each locus was preferentially used. Among the 22 HLA-DR alleles, donors with DRB109:01, DRB104:06, and DRB115:01 showed strong responses (**Figure 1B**). Among the 19 HLA-DQ alleles, DQA1*03:01/DQB1*03:02 showed moderate response (**Figure 1C**). Among the 13 HLA-DP alleles, DPA1*01:03/DPB1*04:02 showed a strong response; DPA1*02:02/DPB1*05:01 and DPA1*02:01/DPB1*17:01 showed a moderate response (**Figure 1D**). In the CMV pp65-specific T cell response, the frequency of the HLA alleles correlated with the proportion of responses (26). However, in the SARS-CoV-2 cross-reactive CD4^+^ T cell response, the proportion of positive responses of each allele was not significantly correlated with the allele frequency of HLA-DR, -DQ, and -DP (**Figure 1E**).

### Allele Dominance in SARS-CoV-2 Cross-Reactive CD4^+^ T Cell Response by an HLA Class II Within Individuals

**Figure 2:**
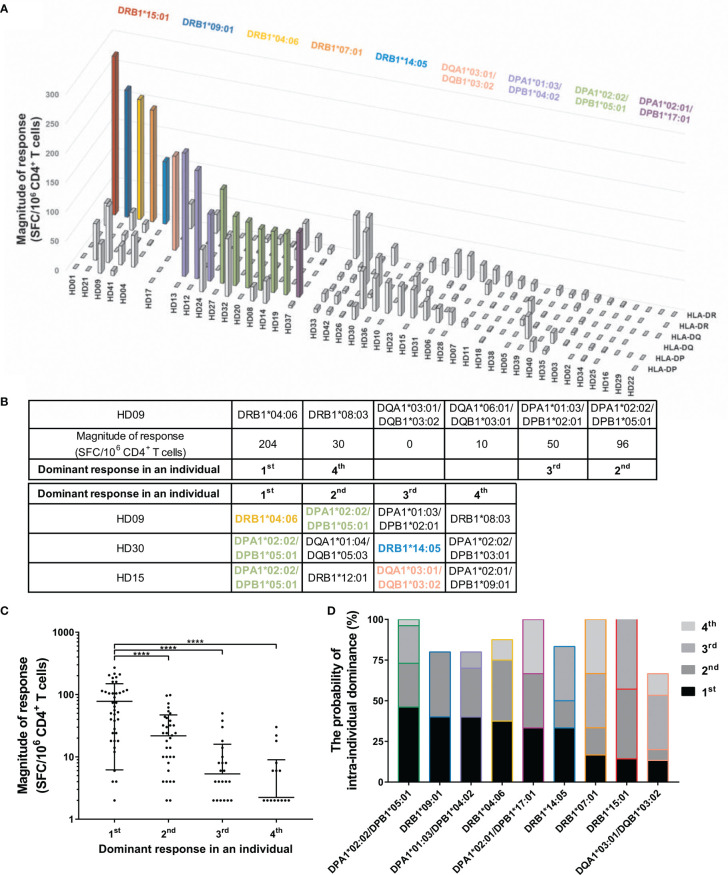
CD4^+^ T cell responses restricted by single HLA class II allotype within individuals. CD4^+^ T cells were stimulated with an allotype-matched aAPCs pulsed with a mixture of peptide pools of spike, nucleocapsid, and membrane protein. **(A)** CD4^+^ T cell responses (vertical) by two allotypes of HLA-DR, -DQ, and -DP loci (depth) in each individual (horizontal). The magnitude of response above 50 SFCs/5 × 10^5^ are colored and the alleles corresponding to the response are shown in the same color. **(B)** A schematic diagram of the analysis of dominant response in individuals. **(C)** The order of highest response by an allotype within an individual is presented as the dominant response in an individual. Each dot represents a response by an allotype. Data are shown as mean ± standard deviation (SD) of the order-specific response. Statistical analysis was performed using one-way ANOVA. ****P < 0.0001. **(D)** The probability of intra-individual dominance presents (the number of donors with the corresponding order from the responses by the allotype)/(the number of donors with the allotype). The donors who did not respond by any allotype were excepted. The order and color of the allele follow **(B)** and **(A)**, respectively.

A heterozygous individual expresses six HLA class II allotypes co-dominantly. To identify the number of allotypes used for the response, we analyzed the SARS-CoV-2 cross-reactive T cells by two allotypes of HLA-DR, -DQ, and -DP loci within individuals. Defining strong and moderate responses as dominant T cell responses, in 16/42 cases (38.1%), unexposed individuals showed a dominant T cell response restricted by one HLA allotype (**Figure 2A**). The remaining 26/42 individuals did not show a dominant T cell response by an HLA allotype. We classified the dominance of HLA allotypes 1–4 in the order of the dominant response in an individual to compare the magnitude of response without an arbitrary threshold (**Figures 2B, C**). The highest response by one allotype was significantly higher than the second, third, and fourth highest responses by the other allotypes within individuals (one-way ANOVA, p <0.0001). The probability of responding first, second, third, and fourth highest within individuals varied even among those alleles that showed a dominant T cell response (**Figure 2D**).

### Antigen Immunodominance in SARS-CoV-2 Antigen-Specific CD4^+^ T Cell Responses Within Individuals

**Figure 3:**
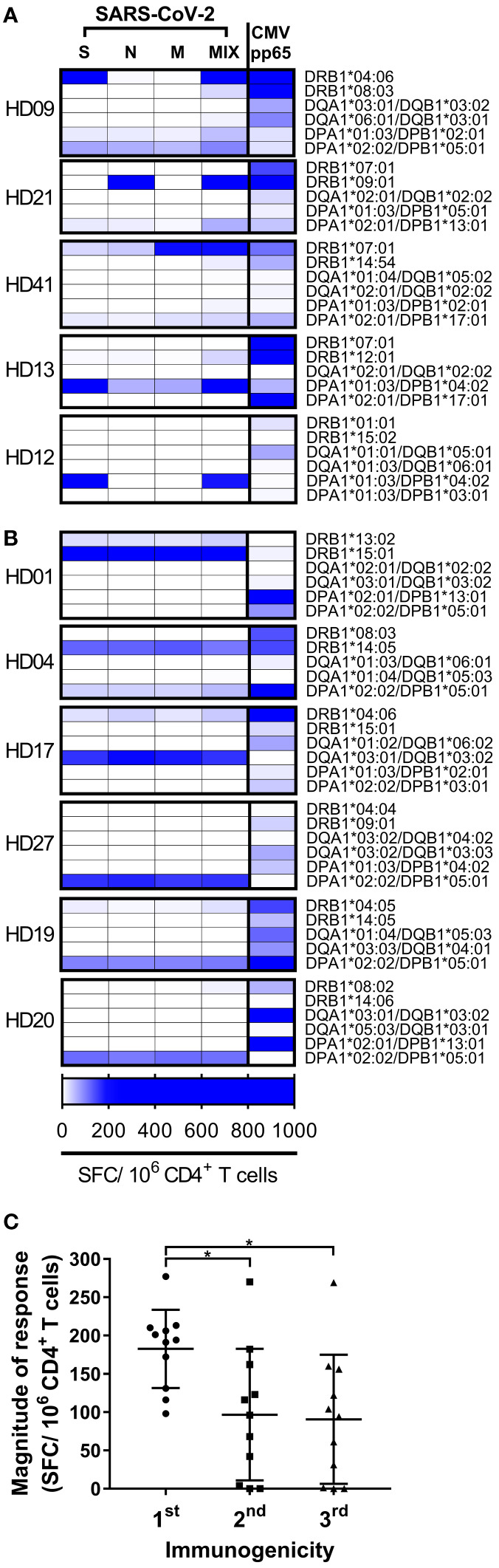
CD4^+^ T cell responses by each HLA class II allotype to each SARS-CoV-2 antigen in each individual. **(A, B)** CD4^+^ T cell responses by an allotype were measured individually with spike (S), nucleocapsid (N), membrane (M), or CMV pp65, and with a mixture (MIX) of peptide pools of S, N, and M protein. High responses are highlighted at the blue end of the spectrum. **(C)** The order of immunogenicity indicates the order of highest response to S, N, or M among the responses by an allele that showed the strong response in an individual. Each dot represents a response by an allotype to a peptide pool of a protein. Data are shown as mean ± SD. Statistical analysis was performed using one-way ANOVA. *P < 0.05.

A few epitopes account for most of the T cell responses to SARS-CoV-2 in a population (29). To determine whether epitopes of the dominant T cells by an allele were distributed in several proteins or one protein, CD4^+^ T cells were stimulated individually by each SARS-CoV-2 peptide pool-loaded aAPCs. Of the 11 donors with dominant T cell responses, five responded primarily to one antigen with an HLA allele (**Figure 3A**). Of the 11 donors with dominant T cell responses, six responded similarly to each antigen with an HLA allele (**Figure 3B**). In the sum of dominant T cell responses by the allele of each individual, the highest response to one protein was significantly higher than the second and third highest responses to the other protein (**Figure 3C**, one-way ANOVA, p = 0.0314 for 1st vs. 2nd, p = 0.0204 for 1st vs. 3rd).

**Figure 4:**
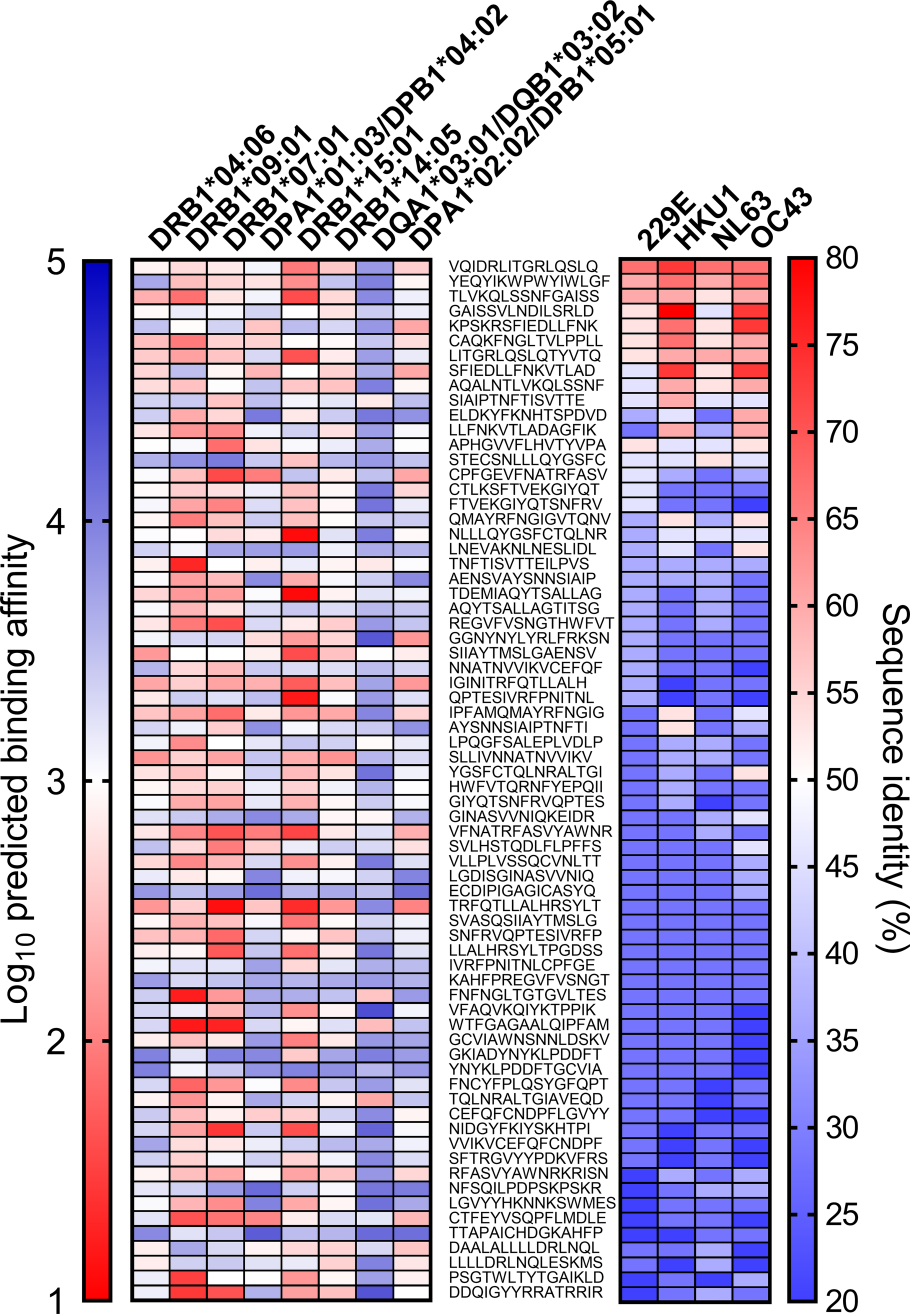
Predicted binding affinity and homology of cross-reactive SARS-CoV-2 epitope. The SARS-CoV-2 epitope sequence that was reported previously (middle) and predicted binding affinity to the allele in **Figures 3** (left). The prediction of binding affinity (IC50) was performed with NetMHCII using the Immune Epitope Database and Analysis Resource (IEDB). Lower binding affinity presented as the red end of the spectrum indicates stronger predicted binding affinity. The sequence similarity of common cold viruses (229E, HKU1, NL63, OC43) to SARS-CoV-2 was expressed as sequence identity (%) and inferred with the epitope conservancy analysis tool (right).

Next, we analyzed predicted half-maximal inhibitory concentration of the cross-reactive SARS-CoV-2 epitopes identified in the other study and aligned with these alleles (19) (**Figure 4**). The alleles that responded primarily to one antigen showed a higher affinity to the cross-reactive epitopes than the alleles that responded similarly to each antigen (**Figure S2A**, p <0.0001). The SARS-CoV-2 epitope recognized in unexposed donors was reported to have high homology to common cold coronaviruses (19). The epitope similarity with common cold coronaviruses (OC43, HKU1, NL63, and 229E) was aligned additionally to the binding affinity of these alleles. The conservancy correlated with higher binding in the DRB1*04:06 and DPA1*01:03/DPB1*04:02 alleles, and the other alleles did not correlate significantly (p = 0.0107 for DRB1*04:06, p = 0.0478 for DPA1*01:03/DPB1*04:02). The cross-reactive epitope discovered in the previous study was mainly a spike protein among spike, nucleocapsid and membrane protein. Considering that the DRB1*04:06 and DPA1*01:03/DPB1*04:02 responded to a spike protein, it is speculated that some alleles respond primarily to one antigen in the case of the high binding affinity or high sequence homology. In total, SARS-CoV-2 cross-reactive-dominant T cells targeted preferentially one protein by one HLA allele within an individual, although there was a deviation in the individual alleles.

### SARS-CoV-2-Specific CD4^+^ T Cell Responses by Combinations of HLA-DP Heterodimer

**Figure 5:**
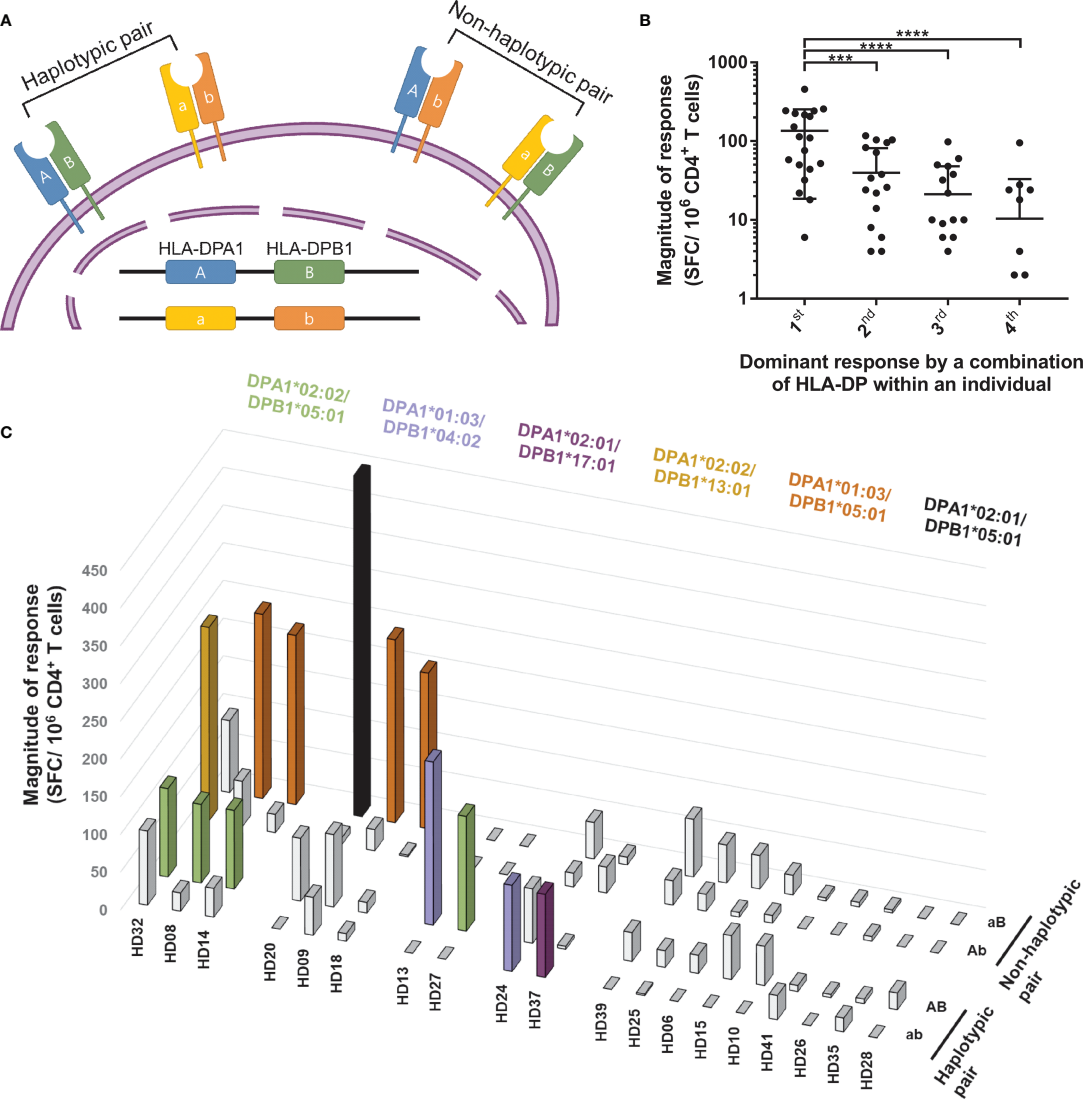
CD4^+^ T cell responses restricted by HLA-DP heterodimer within individuals. CD4^+^ T cells were stimulated with a heterodimer-matched aAPCs pulsed with a mixture of peptide pools of spike, nucleocapsid, and membrane protein. **(A)** Schematic diagram showing two HLA-DP heterodimers encoded by the same allele on DPA and DPB loci (haplotypic pair), the other two HLA-DP heterodimers are encoded by different allele on DPA and DPB loci (non-haplotypic pair). **(B)** Dominant response by a combination of HLA-DP within an individual presents the order of highest response by an HLA-DP heterodimer within an individual. Each dot represents a response by an HLA-DP heterodimer. Data are shown as mean ± SD of 19 individuals. Statistical analysis was performed using one-way ANOVA. ***P <0.001, ****P <0.0001. **(C)** CD4^+^ T cell responses (vertical) by four HLA-DP combinations (depth) in each double heterozygous individual (horizontal). The magnitude of response above 50 SFCs/5 × 10^5^ are colored and the alleles are presented with the same color.

The HLA-DPA locus, encoding the alpha chain of HLA-DP, is polymorphic, and the common HLA-DPA alleles are DPA1*01:03, DPA1*02:02, and DPA1*02:01. DPA1*01:03 accounts for approximately half the HLA-DPA frequency; therefore, most studies did not determine the alpha chain in the peptide binding specificity of HLA-DP. However, in individuals whose HLA-DPA and HLA-DPB are double heterozygous, four combinations of the HLA-DP heterodimers are possible to be expressed and form heterodimers with different alpha chains (**Figure 5A**). To investigate the T cell response patterns for the four HLA-DP combinations, we measured the SARS-CoV-2-specific CD4^+^ T cell responses in 19 double heterozygous individuals (**Figure 5C** and **Table 2**). Three individuals showed dominant T cell responses with two combinations, and seven individuals showed dominant T cell responses with one combination. The remaining nine individuals did not show a dominant T cell response by an HLA-DP combination. The magnitude of responses by the four combinations of HLA-DP was then compared without an arbitrary threshold, as analyzed in **Figures 2B, C**. The highest response by one combination was significantly higher than that of the second, third, and fourth highest responses by the other combinations within individuals (**Figure 5B**, one-way ANOVA, p = 0.0001 for 1st vs. 2nd, p <0.0001 for 1st vs. 3rd and 1st vs. 4th).

**Table 2:** Four HLA-DP combinations in 19 double heterozygous individuals.

Donor	Haplotypic pair		Non-haplotypic pair	
	ab	AB	Ab	aB
	DPA1*/DPB1*	DPA1*/DPB1*	DPA1*/DPB1*	DPA1*/DPB1*
HD32	02:01/13:01	02:02/05:01	02:02/13:01	02:01/05:01
HD08	01:03/04:02	02:02/05:01	02:02/04:02	01:03/05:01
HD14	01:03/02:01	02:02/05:01	02:02/02:01	01:03/05:01
HD20	02:01/13:01	02:02/05:01	02:02/13:01	02:01/05:01
HD09	01:03/02:01	02:02/05:01	02:02/02:01	01:03/05:01
HD18	01:03/02:01	02:02/05:01	02:02/02:01	01:03/05:01
HD13	02:01/17:01	01:03/04:02	01:03/17:01	02:01/04:02
HD27	01:03/04:02	02:02/05:01	02:02/04:02	01:03/05:01
HD24	01:03/04:02	02:02/05:01	02:02/04:02	01:03/05:01
HD37	02:01/17:01	02:02/05:01	02:02/17:01	02:01/05:01
HD39	01:03/02:02	02:02/05:01	02:02/02:02	01:03/05:01
HD25	01:03/02:02	02:02/05:01	02:02/02:02	01:03/05:01
HD06	01:03/04:02	02:02/05:01	02:02/04:02	01:03/05:01
HD15	02:01/09:01	02:02/05:01	02:02/09:01	02:01/05:01
HD10	01:03/04:01	02:02/05:01	02:02/04:01	01:03/05:01
HD41	02:01/17:01	01:03/02:01	01:03/17:01	02:01/02:01
HD26	02:01/14:01	02:02/05:01	02:02/14:01	02:01/05:01
HD35	01:03/04:02	02:02/05:01	02:02/04:02	01:03/05:01
HD28	01:03/04:02	02:02/05:01	02:02/04:02	01:03/05:01

Next, we divided the four combinations into a non-haplotypic pair, which was not maternal and paternal haplotypes, and a haplotypic pair, and analyzed the T cell responses (**Table 2**). We assessed the allotype-specific response at each locus, assuming T cells would respond with the HLA haplotype (**Figure 2**). However, to cytomegalovirus pp65, the dominant T cell responses were restricted by the haplotypic and non-haplotypic pairs (**Figure S3**). For SARS-CoV-2, the dominant T cell responses were by one haplotypic pair and non-haplotypic pair in individuals who showed dominant T cell responses by two combinations (**Figure 5C**). In individuals who showed dominant T cell responses with one combination, three responded with the non-haplotypic pair, and four responded with the haplotypic pair. Overall, antigen-specific T cells responded with an allotype of haplotypic pair and an allotype of non-haplotypic pair.

## Discussion

For SARS-CoV-2, the proportion of strong response by HLA-DR was higher than that by HLA-DQ and -DP (**Figure 1A**). Cytomegalovirus pp65-specific T cell response by HLA-DR was also higher than HLA-DQ and –DP (26). For SARS-CoV-2, the number of epitopes presented by HLA-DR was more than that by HLA-DQ or -DP in convalescent COVID-19 donors (29). The membrane expression of HLA-DR was higher than that of HLA-DQ and -DP in peripheral blood mononuclear cells and aAPCs transduced with a single allele at the same MOI (26, 30), suggesting that HLA-DR-restricted T cells are highly for selection against pathogens. Unlike the response to cytomegalovirus pp65, the SARS-CoV-2-specific T cell response restricted by HLA-DP was significantly higher than that by HLA-DQ, and the magnitude of the response was similar to that by HLA-DR (**Figure 1**). A conserved immunodominant region in the spike protein was restricted by HLA-DR and -DP (31), supporting the high magnitude of response by HLA-DP (**Figures 1A, B, D**).

At the population level, immunodominant epitopes for SARS-CoV-2 are presumed to be promiscuous and capable of binding multiple HLA allelic variants, similar to epitopes for *Mycobacterium tuberculosis* and Dengue virus (32–34). At the individual level, pre-existing SARS-CoV-2-specific T cells responded dominantly to one allotype (**Figures 2A, B** **)**. Moreover, one allotype-restricted T cell responded mainly to one antigen among spike, membrane, and nucleocapsid proteins in 5/11 individuals (**Figure 3A**). The T cell response to pp65 of cytomegalovirus, which has infected most of the world’s population, also showed the allele dominance within an individual (26, 27). In CMV-specific T cell responses, diverse high-affinity clones were stimulated at early time points after infection; however, at late time points, a few low-affinity clones predominated (35, 36). The allele dominance in SARS-CoV-2 cross-reactive CD4^+^ T cells might be the survival of T cells by competing for affinity with viral peptides and HLA allotypes under infection by viruses with sequence homology.

DRB1*07:01 restricted the dominant response in HD41 (**Figure 3A**). In HD21, DRB1*09:01 restricted the dominant response and in HD13, DPA1*01:03/DPB1*04:02 restricted the dominant response and DRB1*07:01 restricted no T cell response although HD21 and HD13 expressed DRB1*07:01. In total, there was a hierarchy among the alleles that caused the dominant T cell response (**Figure 2C**). According to the expression quantitative trait locus analysis from the peripheral blood, different HLA-DR alleles biased the usage of TCR V gene encoding CDR1 and CDR2 (37). In 6/11 individuals, the responses to three different antigens were restricted by an identical HLA allele (**Figure 3B**). In tuberculosis-susceptible mice with the H2-Aj allele, the CDR3α and CDR3β repertoires were more convergent than the H2-Ab allele; therefore, different alleles shape different CDR3 landscapes (38). It is speculated that the TCR repertoire was shaped differently by the combination of the alleles in three loci, and the allele-restricted T cell was more divergent than the other allele-restricted T cell in an individual.

An allele-restricted T cell mainly responded to the spike protein in most individuals (**Figure 3A**). However, there was a dominant T cell response to the nucleocapsid or membrane proteins in an individual. COVID-19 patients with memory T cells specific for the common cold coronavirus developed mild symptoms (21). In addition, individuals who already had spike protein-specific cross-reactive CD4^+^ T cells showed high functional avidities throughout the initiation of the T cell response after SARS-CoV-2 infection (39). The BNT162b2 COVID-19 spike mRNA vaccine boosted the pre-existing SARS-CoV-2 spike cross-reactive T cells. Moreover, an adenovirus-based COVID-19 vaccine encoding both spike and nucleocapsid proteins elicited Th1 dominant responses after a single prime injection (40). A vaccine encoding all the spike, nucleocapsid, and membrane protein might re-activate pre-existing cross-reactive T cells and evoke secondary-like immune responses in more individuals than the only spike-encoding vaccine.

At the population level, the HLA haplotype frequency distribution could be affected by the infection of a pathogen (41, 42). For cytomegalovirus pp65, the frequency of the HLA alleles showed a correlation with the proportion of donors having a T cell response (26). However, for SARS-CoV-2, HLA-DR, -DQ, and -DP allele frequency showed no significant correlation with T cell responses (**Figure 1E**). The frequency of HLA alleles was not correlated with T cell responses because it is a cross-reactive T cell response to the newly emerged SARS-CoV-2, not common cold coronaviruses-specific T cell responses to common cold coronaviruses. In addition, in HLA-DP double heterozygous individuals, the cross-reactive T cells responded by non-haplotypic pairs, which are not associated with HLA-DP haplotype frequency (**Figure 5**). This result supports the heterozygote advantage of HLA (9, 43).

In response to SARS-CoV-2 by HLA-DP, even if the beta chains were identical, CD4^+^ T cells responded to a specific alpha chain-combination (**Figure 5C**). An epitope of bacterial toxin PE38 restricted by specific HLA-DPB did not induce T cell responses in some individuals with HLA-DPB (44). Moreover, mycobacterial peptide-specific T cells were only stimulated by HLA-DPA-matched lymphoblastoid cell lines (45). Therefore, the peptides presented by HLA-DP were determined not only by the beta chains but also by the alpha chains, and T cells were restricted by one combination of HLA-DP to the given peptides.

Although the isolated CD4^+^ T cells cocultured with aAPCs without HLA or antigens did not activate, the magnetic beads used to isolate CD4^+^ T cells can lead to non-specific activation. There is also potential for the up to 3% non-pure cells remaining to act as antigen-presenting cells in this experiment. Limitations of this study include the male predominance (male 90%; female 10%) and the young age group (median age 26).

The cross-reactive SARS-CoV-2-specific CD8^+^ T cell response should be investigated in an allotype-specific manner to understand whether the HLA class I allele-restricted response is akin to the HLA class II allele-restricted response. This study is not powered to substantiate single-allotype-dominant response in SARS-CoV-2 infection. We plan a chronological study following SARS-CoV-2 infection and vaccination to probe whether the single-allotype dominance is a general phenomenon in T cell response to viral infection. Allotype-dependency of CD4^+^ responses to common cold coronaviruses should also be investigated how that differs from the cross-reactive responses to SARS-CoV-2. The alleles that showed a dominant response in **Figure 3** were analyzed with their predicted binding affinity and conservancy of the epitopes in **Figure 4**. However, the magnitude of response by these alleles or proportion of positive response did not correlate with binding affinity or the number of predicted binding epitopes (**Figures S2B–E**). The cross-reactive epitopes and their HLA restriction should be defined in further study.

In summary, pre-existing cross-reactive CD4^+^ T cell response to SARS-CoV-2 was high for some HLA class II allotypes and loci. The pre-existing SARS-CoV-2-specific T cells were restricted by one allotype among HLA-DR, -DQ, and -DP allotypes within an individual, and the T cells responded greatly to one SARS-CoV-2 antigen among the spike, membrane, and nucleocapsid proteins. One combination among the four HLA-DP combinations restricted the dominant T cell response, and non-haplotypic pairs also restricted this response in HLA-DP double heterozygous individuals. These results shed light on allele dominance in cross-reactive T cell responses to SARS-CoV-2, which have implications for understanding different responses to the COVID-19 vaccine and the clinical course of COVID-19.

## Data Availability Statement

The datasets presented in this study can be found in online repositories. The names of the repository/repositories and accession number(s) can be found below: https://www.ncbi.nlm.nih.gov/bioproject/PRJNA721949/.

## Ethics Statement

The studies involving human participants were reviewed and approved by the Institutional Review Board of the Catholic University of Korea (MC21SASI0009). The patients/participants provided their written informed consent to participate in this study.

## Author Contributions

Y-SH and T-GK conceived and designed the experiments. I-CB typed the HLA. Y-SH, Y-HL, S-MK, H-AJ, and H-JS contributed to sample preparation. Y-SH carried out the experiments. Y-SH and T-GK analyzed data and wrote the manuscript. T-GK supervised the project. All authors contributed to the article and approved the submitted version.

## Funding

This study was supported by a grant of the Korea Health Technology R&D Project through the Korea Health Industry Development Institute (KHIDI), funded by the Ministry of Health & Welfare, Republic of Korea (HI14C3417).

## Conflict of Interest

The authors declare that the research was conducted in the absence of any commercial or financial relationships that could be construed as a potential conflict of interest.

## Publisher’s Note

All claims expressed in this article are solely those of the authors and do not necessarily represent those of their affiliated organizations, or those of the publisher, the editors and the reviewers. Any product that may be evaluated in this article, or claim that may be made by its manufacturer, is not guaranteed or endorsed by the publisher.
