# Supplementary material for: Comprehensive Analysis of CD4+ T Cell Response Cross-Reactive to SARS-CoV-2 Antigens at the Single Allele Level of HLA Class II
Source: Front Immunol. 2022 Jan 6;12:774491. doi: 10.3389/fimmu.2021.774491 (PMC8770530; doi:10.3389/fimmu.2021.774491)
Supplement: Supplementary file 1 [file DataSheet_1.pdf]

Supplementary Material

1 Supplementary Figures and Tables

Supplementary Figures

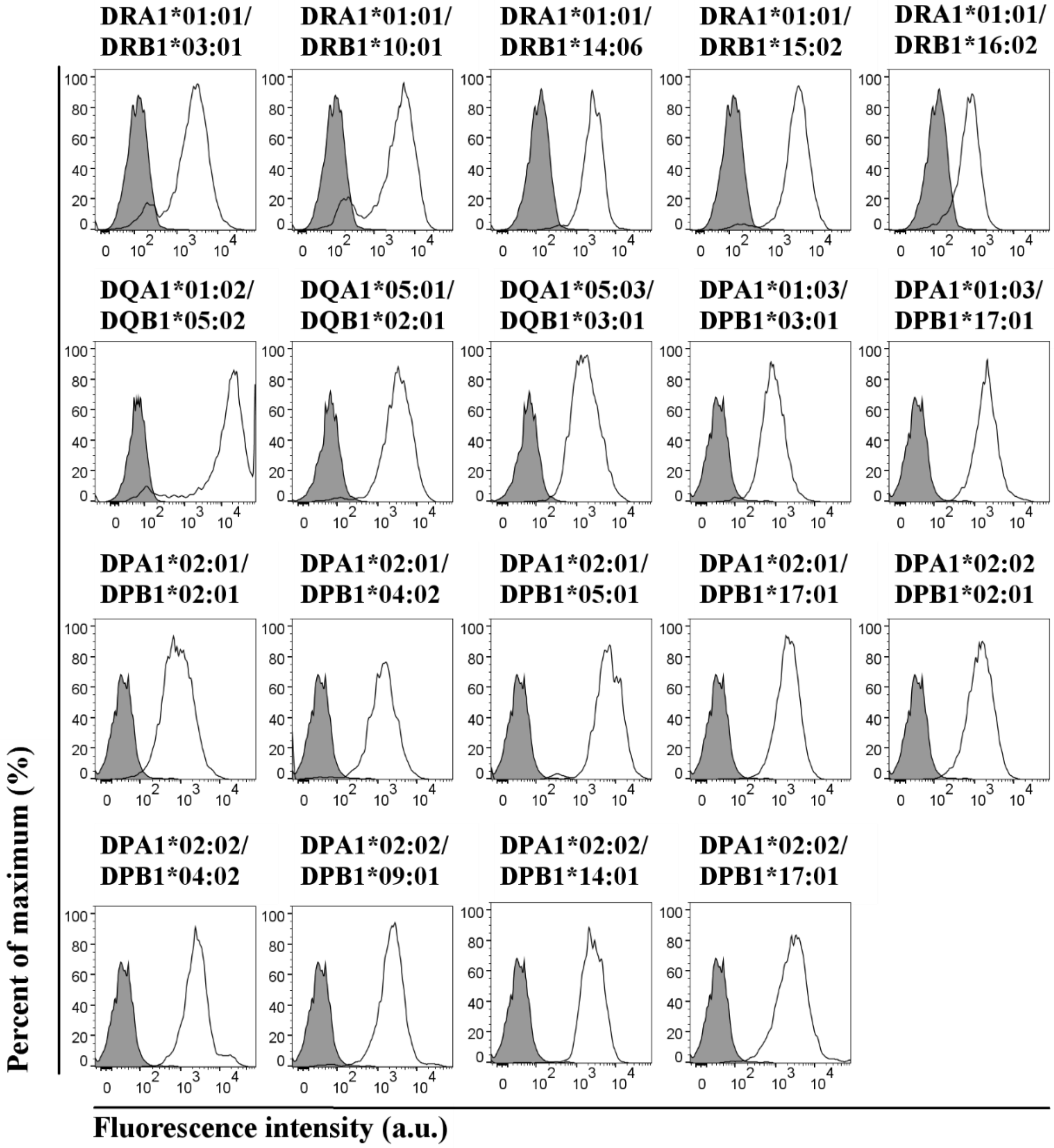

Fig. S1. Expression of HLA-DR, DQ and -DP on single HLA-DR, -DQ and -DP allotype-specific aAPCs.

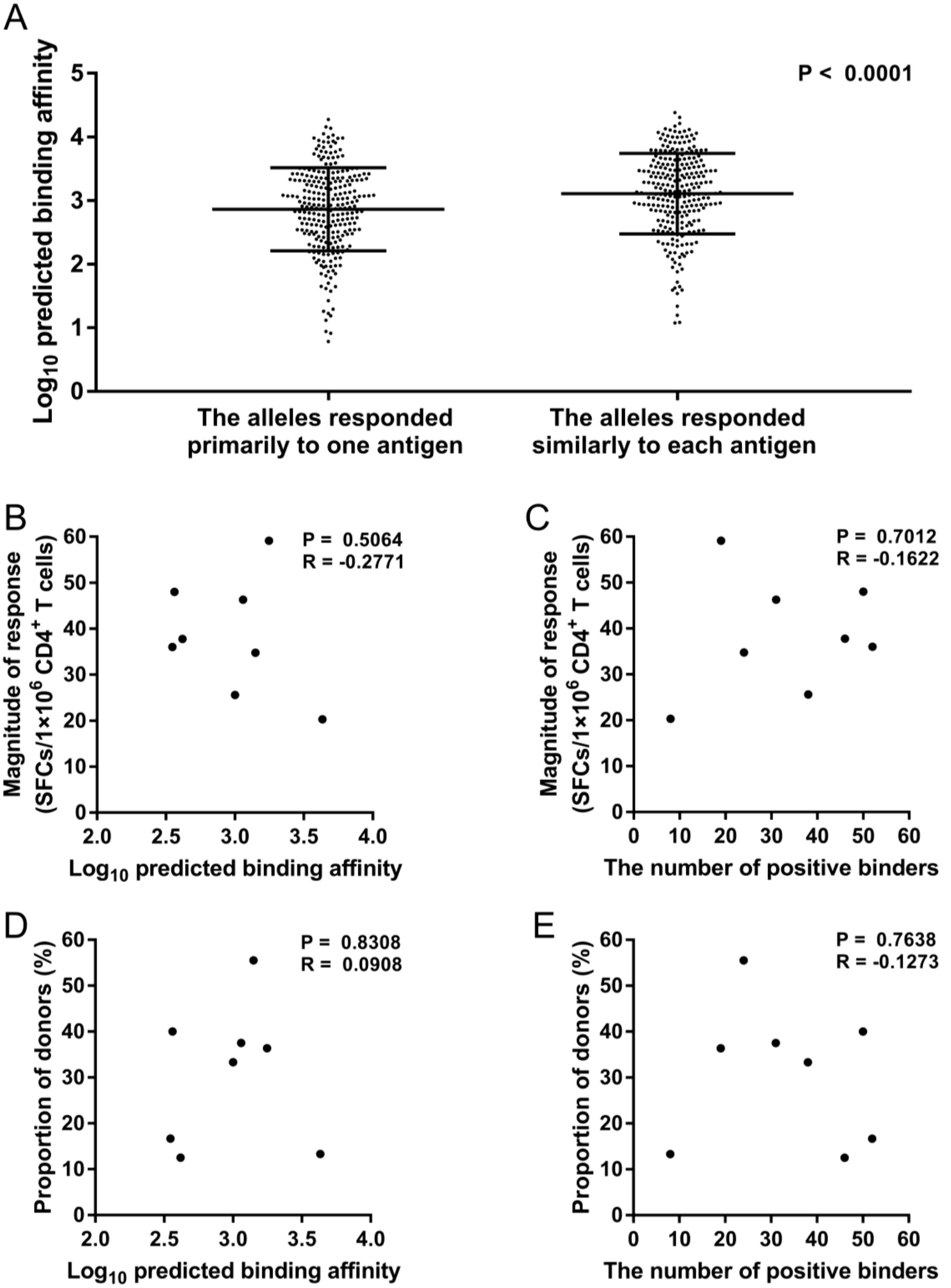

Fig. S2. The analysis of predicted binding affinity and correlation with the response by the allotypes. (A) Predicted binding affinity to the epitopes between the alleles that responded primarily to one antigen and the alleles that responded similarly to each antigen were analyzed with an unpaired *t*-test. (B-E) Pearson correlation between the magnitude of response, the positive-response proportion of donors with the allele, predicted binding affinity and the number of positive binders with the IC50 predicted values < 1000 nM (log10 scale < 3).

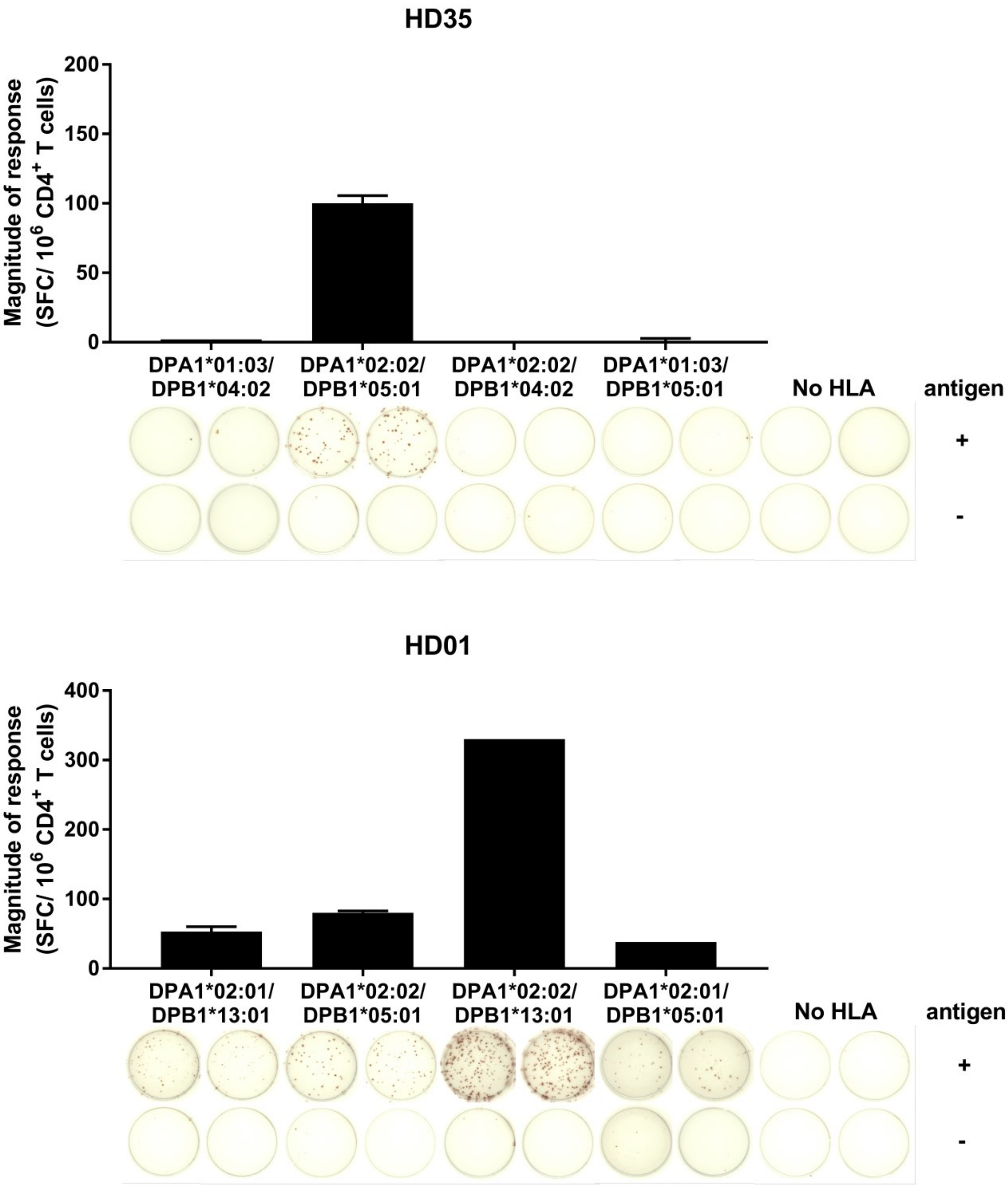

Fig. S3. CMV pp65-specific CD4+ T cell response restricted by an HLA-DP heterodimer in HD35 and HD01. Results are shown as a mean ± SD of duplicates.

Supplementary Tables

Table S1. Primer sequences

ATG GCC ATA AGT GGA GTC CCT	DRA-Forward
TTA CAG AGG CCC CCT GCG	DRA-Reverse
ATG GTG TGT CTG AAG TTC CCT G	DRB1-Forward
ATG GTG TGT CTG AAG CTC CCT	DRB1-Forward
ATG GTG TGT CTG AGG CTC CC	DRB1-Forward
TCA GCT CAG GAA TCC TGT TGG	DRB1-Reverse
TCA GCT CAG GAA TCC TCT TGG	DRB1-Reverse
TCA CCT TCC TCT TGG CTG AAG	DRB1-Reverse
CTT GGG AAG ARG ATG ATC CTA AAC AAA	DQA1-Forward
TTA CCT TCY HTT YCA GGA TGG GAT TCA CAA	DQA1-Reverse
TTT CCC TTY GTC TCA RTT ATG TCT TGG AA	DQB1-Forward
TTA AAA YAG TCT CAG GAG TCA GTG CAG	DQB1-Reverse
ATG CGC CCT GAA GAC AGA	DPA1-Forward
CAC CTT TAC AGT ATT TCA CAG GG	DPA1-Reverse
TCC ATG ATG GTT CTG CAG GTT TCT	DPB1-Forward
TTA TGC AGA TCC TCG TTG AAC TTT	DPB1-Reverse

Table S2. Antibodies and reagents

APC Mouse Anti-Human HLA-DR	BD Bioscience	Cat# 559866
Alexa Fluor® 647 Mouse Anti-Human HLA-DQ	BD Bioscience	Cat# 564806
FITC Mouse Anti-Human HLA-DR, DP, DQ (only for DQA1*01:03/DQB1*06:01 and DQA1*01:04/DQB1*05:02 aAPCs)	BD Bioscience	Cat# 555558
Anti-Human HLA-DP (MHC Class II) Monomorphic – DyLight® 650	Leinco Technologies	Cat# H1586
CD4 MicroBeads, human	Miltenyi Biotec	Cat# 130-045-101
PepMix™ SARS-CoV-2 (Spike Glycoprotein)	JPT Peptide Technologies	PM-WCPV-S
PepMix™ SARS-CoV-2 (NCAP)	JPT Peptide Technologies	PM-WCPV-NCAP
PepMix™ SARS-CoV-2 (VME1)	JPT Peptide Technologies	PM-WCPV-VME
PepMix™ HCMVA (pp65) (>90%)	JPT Peptide Technologies	PM-PP65-2
Human IFN-γ ELISPOT Set	BD Bioscience	Cat# 551849
AEC Substrate Set	BD Bioscience	Cat# 551951
